# CRISPR/Cas9 Guided Mutagenesis of *Grain Size 3* Confers Increased Rice (*Oryza sativa* L.) Grain Length by Regulating Cysteine Proteinase Inhibitor and Ubiquitin-Related Proteins

**DOI:** 10.3390/ijms22063225

**Published:** 2021-03-22

**Authors:** Babar Usman, Neng Zhao, Gul Nawaz, Baoxiang Qin, Fang Liu, Yaoguang Liu, Rongbai Li

**Affiliations:** 1 State Key Laboratory for Conservation and Utilization of Subtropical Agro-Bioresources, College of Agriculture, Guangxi University, Nanning 530004, China; babarusman119@gmail.com (B.U.); nengzhao@st.gxu.edu.cn (N.Z.); gulnawazmalik@yahoo.com (G.N.); bxqin@gxu.edu.cn (B.Q.); fangliu@gxu.edu.cn (F.L.); 2 State Key Laboratory for Conservation and Utilization of Subtropical Agricultural Bioresources, South China Agricultural University, Guangzhou 510642, China

**Keywords:** rice, genome editing, homozygous, yield, proteomics

## Abstract

Clustered Regularly Interspaced Short Palindromic Repeats (CRISPR)/CRISPR-associated protein (Cas9)-mediated genome editing has become an important way for molecular breeding in crop plants. To promote rice breeding, we edited the *Grain Size 3* (*GS3*) gene for obtaining valuable and stable long-grain rice mutants. Furthermore, isobaric tags for the relative and absolute quantitation (iTRAQ)-based proteomic method were applied to determine the proteome-wide changes in the *GS3* mutants compared with wild type (WT). Two target sites were designed to construct the vector, and the Agrobacterium-mediated method was used for rice transformation. Specific mutations were successfully introduced, and the grain length (GL) and 1000-grain weight (GWT) of the mutants were increased by 31.39% and 27.15%, respectively, compared with WT. The iTRAQ-based proteomic analysis revealed that a total of 31 proteins were differentially expressed in the *GS3* mutants, including 20 up-regulated and 11 down-regulated proteins. Results showed that differentially expressed proteins (DEPs) were mainly related to cysteine synthase, cysteine proteinase inhibitor, vacuolar protein sorting-associated, ubiquitin, and DNA ligase. Furthermore, functional analysis revealed that DEPs were mostly enriched in cellular process, metabolic process, binding, transmembrane, structural, and catalytic activities. Pathway enrichment analysis revealed that DEPs were mainly involved in lipid metabolism and oxylipin biosynthesis. The protein-to-protein interaction (PPI) network found that proteins related to DNA damage-binding, ubiquitin-40S ribosomal, and cysteine proteinase inhibitor showed a higher degree of interaction. The homozygous mutant lines featured by stable inheritance and long-grain phenotype were obtained using the CRISPR/Cas9 system. This study provides a convenient and effective way of improving grain yield, which could significantly accelerate the breeding process of long-grain japonica parents and promote the development of high-yielding rice.

## 1. Introduction

Rice is one of the most important crops globally and a staple food for more than half of the world’s population. Increasing rice production plays an extremely important role in the stability of the world economy and the development of human society. Since the 1960s, China’s rice yield has been stagnant for a long time after two major leaps in dwarf and hybrid rice breeding. At present, with population increase, arable land reduction, environmental pollution, and frequent extreme weather disasters, rice production is facing severe challenges [1]. It is urgent to apply new technologies to break the bottleneck of rice production. Rice yield is a complex agronomic trait mainly determined by the effective panicle number (PN), grain number per panicle (GNPP), and 1000-grain weight (GWT), all of which are typical quantitative traits. In recent years, the demand for high-yield and quality rice has increased, especially for long-grain rice. In the process of traditional rice variety improvement, the aggregation of excellent genes is mainly achieved through hybridization and backcrossing. However, conventional breeding has the disadvantages of a long breeding cycle and low efficiency. Through the development of specific gene chips and functional molecular markers, the function of yield-related genes has been extensively explored, and rice breeding is planned to cultivate new rice varieties with high and stable yields [2]. The completion of rice genome sequencing, development of functional genomics, proteomics, bioinformatics, and the widespread use of next-generation molecular marker technology has laid an important foundation for rice yield improvement [3]. Recently developed gene-editing technologies can bring new improvements to the rapid development of rice varieties with improved grain yield.

Mutants are important materials for studying gene function as well as for breeding programs. Traditional artificial mutations are usually produced by ethyl methanesulfonate (EMS) mutagenesis, radiation mutagenesis, and (transgene-DNA) T-DNA insertion. However, these methods cause random mutations genome wide, which are difficult to detect and sometimes deleterious. Through transgenic technologies, genetically modified (GM) crops can be given a variety of beneficial traits, such as insect resistance, herbicide tolerance, stress resistance, improved nutritional value, and grain yield. There is still a big gap between GM rice and large-scale commercialization. The technical reason is that it is difficult to completely remove the T-DNA fragments. At present, the Clustered Regularly Interspaced Short Palindromic Repeats/CRISPR-associated protein 9 (CRISPR/Cas9) emerged as a new generation of gene-editing technology, and it is widely used in crop breeding and functional genomics research [4,5,6,7,8,9]. Recent studies have proved that CRISPR/Cas9 can generate T-DNA-free mutants with inheritable mutations [10,11,12].

Researchers generally believe that grain length (GL), grain width (GWD), and aspect ratio are generally controlled by multiple genes. Studies have also shown that the genes that control various traits of grain type also have complementary and additive effects [13]. Many quantitative trait loci (QTLs) related to grain size have been identified in rice, such as the negative regulatory factors *GW2*, *GS9*, *qW5/GW5*, and *TGW6* [14,15,16,17,18], and positive regulators including *GL2*, *GL3.1*, *GS5*, *GL7*, *GLW7*, and *GW8* [19,20,21,22]. *Grain Size 3* (*GS3*) is the first gene to be cloned to regulate grain yield, and its loss-of-function mutations result in enhanced grain yield [15,23]. The negative regulator genes related to grain yield are suitable for carrying out knockout offspring to obtain slender kernels. It is difficult to increase yield through traditional breeding methods, so the employment of modern technologies is necessary to achieve the required grain production in a timely manner. Currently, rice is one of the most successful crops for CRISPR/Cas9 applications. Studies have shown that through CRISPR/Cas9-mediated genome editing, the probability of obtaining homozygous or biallelic genes is as high as 90% in T_0_ generation [24]. Previous studies suggest that mutations in the *GS3* gene results in increased GL and GWT, and the overall yield improvement [25]. Zhao et al. (2018) [16] used CRISPR/Cas9 technology to generate mutations in the first exon of *GS9*, which resulted in a frameshift mutation, disrupted *GS9* normal expression, and increased GL. Lowder et al. (2015) [26] designed sgRNAs mediated by U6 and U3 promoters respectively according to the sequences of rice target genes *OsYSA* and *OsROC5* and obtained albino seedling and curled leaf phenotype. CRISPR/Cas9-based mutations in three homoeologs of cytochrome *P450* genes and *OsBADH2* resulted in increased grain yield and aroma [10]. After *OsPYL9* was mutated by CRISPR/Cas9, the drought tolerance and grain yield of rice were improved significantly [11]. A recent study has shown that CRISPR/Cas9 can successfully introduce homozygous mutations in *GW8*, resulting in increased grain yield [12]. These studies suggest that CRISPR/Cas9 technology has set a precedent in rice gene editing, and at the same time, it also provides a new technical approach and method for yield improvement.

With the rapid development of proteomics, quantitative study of protein changes has become one of the important contents of proteomics research. Relative and absolute quantitative isotope labeling (iTRAQ) technology combined with tandem mass spectrometry and multidimensional liquid chromatography is the latest technology. The tools for qualitative and quantitative protein research with better effects have been widely used in the proteomics research of rice [10,11,12]. Recently, iTRAQ technology has been used to compare the proteomic changes of CRISPR/Cas9 rice mutants, and differentially expressed proteins (DEPs) have been successfully screened [27,28,29,30,31]. These studies suggest that iTRAQ technology is helpful to study the changes in plant protein expression because of mutations. Therefore, with the continuous improvement of genome and plant protein databases, there will be more room for the application of iTRAQ technology in CRISPR/Cas9 generated mutant plants.

In this study, using CRISPR/Cas9 technology, *GS3* mutants were successfully generated, and comparative proteomic analysis was performed to reveal the changes proteome-wide. Mutants exhibited increased grain size without any change in other agronomic traits. Proteomics screening found that multiple identified proteins were differentially regulated, and mutant plants showed enhanced grain yield. In short, this work suggests that *GS3* mutants hold great potential in rice breeding to improve grain yield.

## 2. Results

### 2.1. Construction of CRISPR/Cas9 Expression Vector

**Figure 1:**
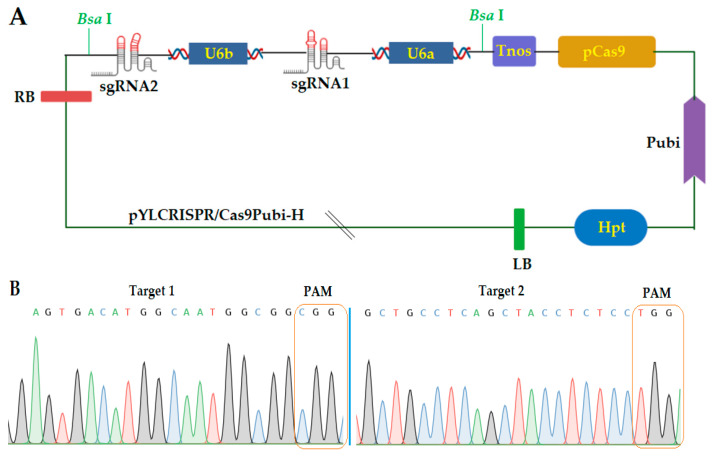
(**A**) Schematic representation of pYLCRISPR/Cas9 vector construction and (**B**) sequencing peak map of both target sites assembled in vector. sgRNA, single guided RNA; LB, left border; mpCas9, Cas9 protein; U6, rice U6 promoter; HPT, hygromycin phosphotransferase gene; Bsa, cutting sites; Pubi, maize ubiquitin promoter; Tnos: gene terminator, RB, right border; PAM, protospacer adjacent motif.

According to the “Golden Gate” cloning method, the guided RNA (gRNA) expression cassette with two targets was connected to the pYLCRISPR/Cas9Pubi-H vector backbone (Figure 1A). The amplification of the sgRNA expression cassette for the first and second targets (T1 and T2) was verified by the overlapping polymerase chain reaction (PCR). The CRISPR/Cas9 binary vector was effectively built, and both sgRNA sequences were confirmed in the vector (Figure 1B) by using the SP-L1 and SP-R (Table S1) primers.

### 2.2. Obtaining Mutant Plants and Genotyping

In total, we treated 75 calli with transformed *A. tumefaciens* and attained 20 plantlets. We extracted the corresponding genomic DNA from each mutant plant, and the specific primers *HPT*F/R (Table S1) were used to identify whether the T-DNA regions were successfully integrated. The results showed that 15 tissue cultured plantlets were transgenic positive.

Sequencing results revealed that among the 15 plantlets, 12 plantlets were edited, representing an editing efficiency of 80%. According to the decoding of sequencing results, four types of plantlets were obtained with no editing, homozygous editing, monoallelic heterozygous editing, and biallelic heterozygous editing. By counting the different types of edits, we found that there were four WT, four homozygous, three biallelic heterozygous, and four monoallelic heterozygous plantlets for the first target in T_0_ generation. The editing results of the second target revealed that there were three WT, five homozygous, two biallelic heterozygous, and five mono-allelic heterozygous plantlets in the T_0_ generation. 

**Figure 2:**
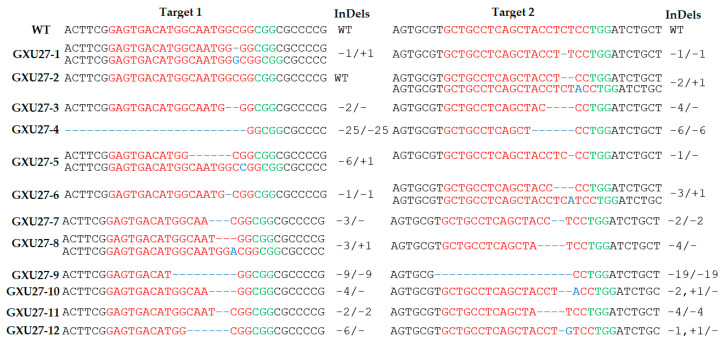
Sequence alignment of both target sites and information about deletions and insertions in all T_0_ mutant lines. The targeted sequence is highlighted in red, and the protospacer adjacent motif (PAM) sequence in green. Deletions and insertions are represented by blue hyphens and uppercase letters, respectively.

Two mutant lines (GXU27-4 and GXU27-9) showed homozygous mutations for both target sites. GXU27-4 exhibited 25 bp and 6 bp deletions at the first and second target positions, respectively. GXU27-9 presented 9 bp and 19 bp deletions at the first and second target locations, respectively (Figure 2). Deletion and insertions with at least one nucleotide were achieved successfully.

Using the Cas9F/R specific primers (Table S2), we amplified the DNA of 20 T_1_ mutant plants for the five most likely positions with the higher off-target ranking. The sequencing results revealed that there were no off-target effects found against sgRNA1 and sgRNA2 in selected putative loci (Table S3).

### 2.3. Screening of T-DNA-Free Mutant Plants in the T_1_ Generation and Segregation Analysis

To obtain mutant plants without T-DNA components, the progeny of the T_0_ generation was evaluated. A total of 27 mutants of the T_1_ generation were screened for T-DNA fragments using Cas9F/R primers. Those mutants that were amplified to the 600 bp fragment length were considered T-DNA positive, whereas mutants not amplified were considered T-DNA-free. Results showed that 15 plants were not amplified to Cas9-specific primers (Figure S1). The T-DNA-free mutants appeared with a frequency of 60%.

Transmissions of the targeted mutations induced by CRISPR/Cas9 were investigated by the self-fertilization of T_0_ mutants and subjected to segregation analyses. The T_1_ progeny of homozygous plants (GXU27-4) exhibited homozygosity for both target sites with the same mutations. These results indicate that homozygous mutations were stably transmitted from T_0_ to T_1_ generation for all target sites. We observed the segregation pattern of a monoallelic heterozygous mutation in GXU27-3 for the first target site and GXU27-8 for the second target site. The T_1_ progeny (GXU27-3 and GXU27-8) of T_0_ monoallelic heterozygous lines was segregated according to Mendelian inheritance and resulted in homozygous and heterozygous mutations and WT plants. The inheritance pattern of biallelic mutations was studied using GXU27-1 for the first target and GXU27-2 for the second target. The progeny of the biallelic mutants also followed the classic Mendelian inheritance (1:2:1) (Table S4). Therefore, gene-edited plants that do not contain T-DNA components can be obtained in the T_1_ generation, and the mutation characteristics of these plants remain consistent with their T_0_ generation.

### 2.4. Investigation of Agronomic Traits

**Figure 3:**
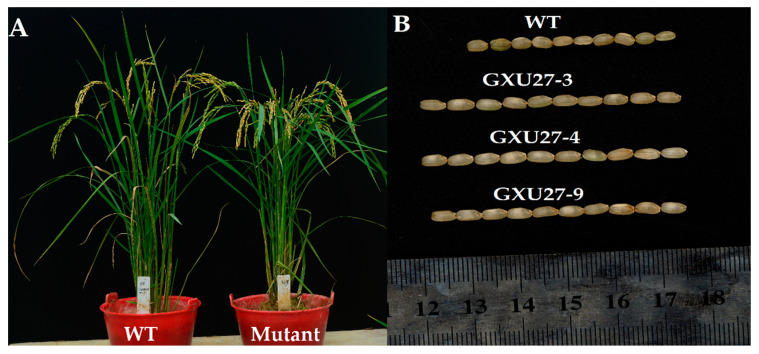
(**A**) Plant type and (**B**) grain phenotype of wild type (WT) and mutants (GXU27-3, GXU27-4, and GXU27-9) in the T_1_ generation.

Two homozygous (GXU27-4 and GXU27-9) and one monoallelic heterozygous line (GXU27-3) from T_0_, T_1_, and T_2_ generations were tested for agronomic traits evaluation. The results showed that the grain size of all mutants was significantly increased than that of the WT, whereas there was not any change in other agronomic traits was observed (Table 1; Figure 3). The GL of mutants was increased from 8.6 to 11.3 mm compared with WT plants. The results showed that the GWT of the mutants was also increased significantly compared with the corresponding WT plants. At the same time, the plant height (PH), PN, flag leaf length (FLL), flag leaf width (FLW), GNPP, and GWD of all *GS3* mutants showed a non-significant difference compared to WT (Table 1). The T_1_ and T_2_ generation showed consistent results with the T_0_ generation, which clearly showed that mutations were passed to the next generation successfully.

### 2.5. Proteomic Data Outcome

**Figure 4:**
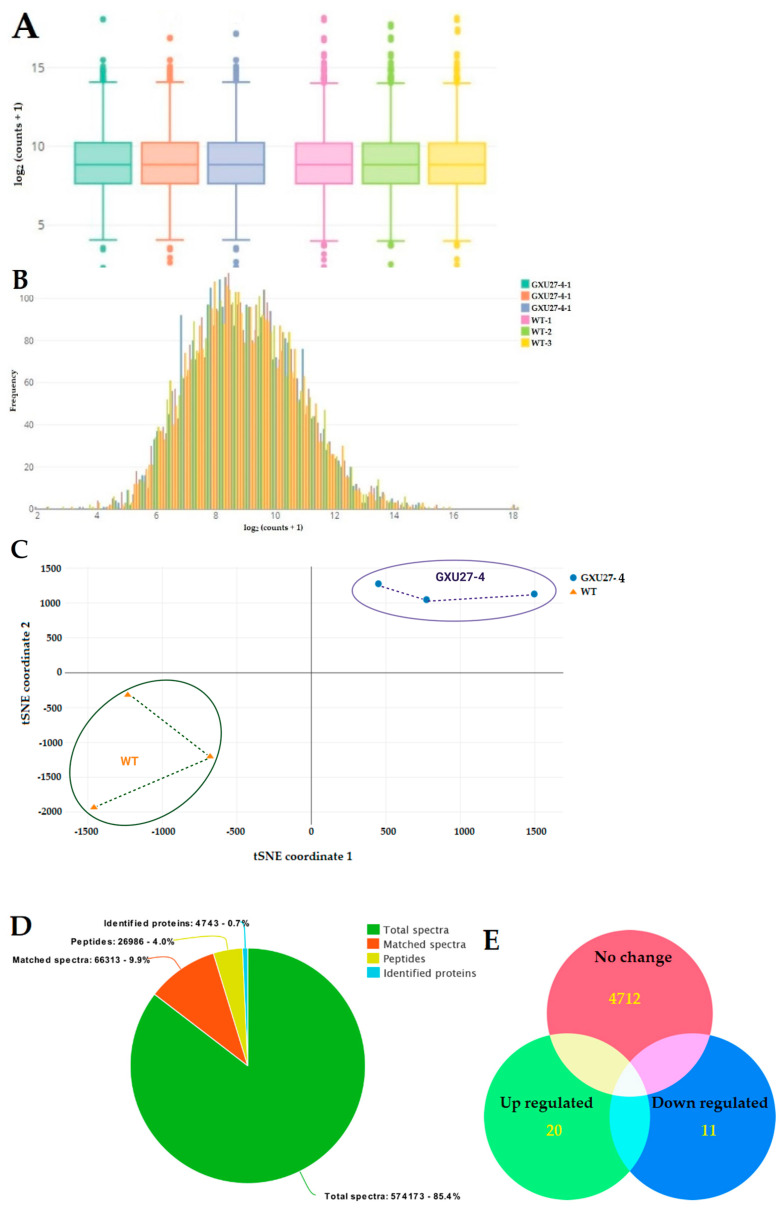
Basic information about the outcome from the proteomic analysis. (**A**) Representation of count data distribution with log2 values in a Box and Whisker plot. (**B**) Histogram showing the frequency of count data distribution of transformed data (**C**) Two-dimensional t-Distributed Stochastic Neighbor Embedding (t-SNE) graph representing the statistical difference between wild type (WT) and mutant line (GXU27-1) (**D**) Information about total spectra, identified matched spectra, peptide, and identified proteins and (**E**) Venn diagram showing the distribution of differentially expressed proteins (DEPs).

The box and whisker plot showed a clear difference between WT and mutant plants’ proteomic data (Figure 4A). The count data distribution plot also showed a clear difference among WT and mutant plants’ expression data, whereas there was no difference between the replicates (Figure 4B). The significant difference between WT and GXU27-1 was found in two-dimensional t-Distributed Stochastic Neighbor Embedding (t-SNE) graph. The points representing the replicates from both samples were near to each other while there was a significant difference among the points sample point (Figure 4C). Using iTRAQ labeled proteomics, 574,173 total spectra, 66,313 matched spectra, and a total of 26,986 peptides were detected in the six samples tested. Peptides were searched through the UniProt database, and finally, 4743 proteins were identified and quantified (Figure 4D; Supplementary File S1). 

The results of differential analysis of protein expression levels showed that there were 20 proteins up-regulated and 11 proteins down-regulated, and 4712 proteins were not differentially expressed (Figure 4E). The proteins related to cysteine synthase (A2ZMY2, Q5JNB0, and B8AJV7) cysteine proteinase inhibitor (Q0JNR2, A0A0A7EQF3, and P20907), ubiquitin (A2XEA1), Vacuolar protein sorting-associated protein (Q10NQ3, A2 × 377, A0A0E0GXY5, and Q8H8K1), DNA damage-binding protein 1 (Q6L4S0), DNA ligase (Q7XD67), and some other were differentially regulated (Table 2; Supplementary File S1). 

### 2.6. Functional Assignment and Pathway Analysis

**Figure 5:**
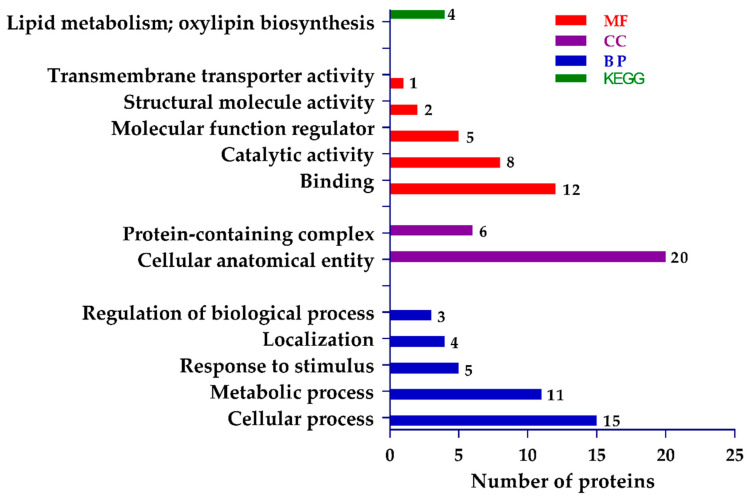
Gene ontology (GO) classification and pathway assignment of differentially expressed proteins (DEPs).

The results of the significant enrichment of gene ontology (GO) function and Kyoto Encyclopedia of Genes and Genomes (KEGG) pathway analysis are shown in Figure 5. GO analysis is mainly divided into three parts: molecular function (MF), biological process (BP), and cellular component (CC). It is often used to provide functional classification labels of DEPs and background knowledge of gene function research.

The results of significant GO enrichment showed that from the perspective of BP, the DEPs were significantly enriched (*p*-value ≤ 0.05) in the cellular process, metabolic process, response to stimulus, localization, and regulation of the BP. Regarding CC, the GO functional attributes were significantly enriched in cellular anatomical entity and the protein-containing complex. Finally, regarding the MF perspective, the DEPs were significantly enriched in binding, catalytic activity, molecular function regulator, structural molecule activity, and transmembrane transporter activity. The result of significant enrichment of the KEGG pathway showed that DEPs were only enriched in lipid metabolism and oxylipin biosynthesis.

### 2.7. Functional Interaction Networks of the Differentially Expressed Proteins (DEPs)

**Figure 6:**
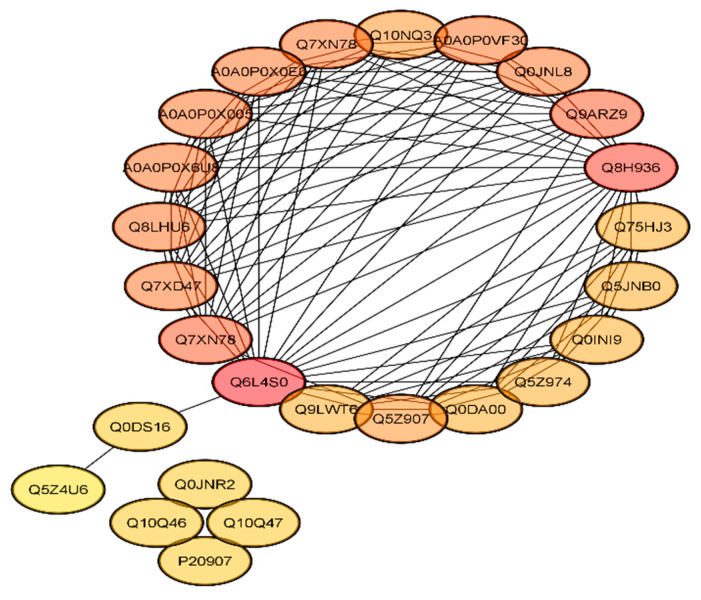
Protein–protein interaction (PPI) network of differentially expressed proteins (DEPs). The nodes with redder color showed a higher degree of interaction, whereas the nodes out of the circle showed less or no interaction.

The Search Tool for the Retrieval of Interacting Genes/Proteins (STRING) database was used to find the protein interactions. After retrieving proteins with the highest connectivity from the projected network, higher interaction was found between Q6L4S0 (DNA damage-binding protein 1), Q8H936 (JUN-activation-domain-binding protein 1), Q7XN7 (ubiquitin-40S ribosomal protein S27a-2), Q9ARZ9 (ubiquitin-40S ribosomal protein S27a-1), Q7XD47 (putative ubiquitin/ribosomal protein S27a fusion protein), A0A0P0X6U8 (putative ubiquitin/ribosomal protein CEP52), A0A0P0X005 (ubiquitin family protein), A0A0P0X0E0 (pentameric polyubiquitin-like), Q7XN78 (polyubiquitin 3 Ubiquitin-related Ubiquitin, and A0A0P0VF30 (polyubiquitin) (Figure 6) with a degree higher than 12 (Supplementary File S1). Some proteins including P20907 (cysteine proteinase inhibitor 2), Q0JNR2 (cysteine proteinase inhibitor 12), Q10Q47 (putative cysteine proteinase inhibitor 7), Q10Q46 (cysteine proteinase inhibitor 6), Q0DS16 (PP1/PP2A phosphatases pleiotropic regulator PRL1), and Q5Z4U6 (putative C2H2 zinc-finger protein) showed a degree value of less than 3 and no or poor interaction with other proteins. The above results revealed that the DNA-damage binding proteins and ubiquitin ribosomal proteins were found to be highly interactive, whereas cysteine proteinase inhibitor proteins showed poor or no interaction.

### 2.8. Verification of mRNA Expression Patterns of GS3 and Differentially Expressed Proteins (DEPs)

**Figure 7:**
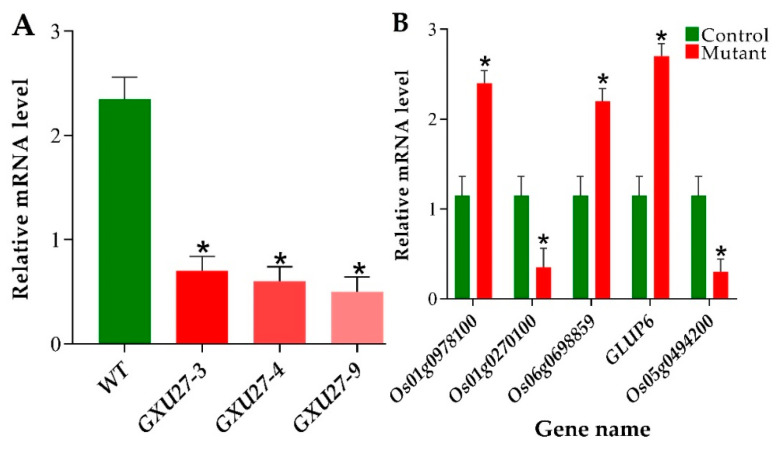
RT-qPCR-based assessment of *GS3* expression and validation proteomic experiment. (**A**) The expression level of *GS3* in wild-type (WT) and mutant plants. (**B**) Expression analysis of five selected genes associated with differentially expressed proteins (DEPs). * Denotes a significant difference, Student’s *t*-test, *p* ≤ 0.01, *n* = 3.

The mRNA expression patterns of *GES3* and five representative DEPs coding genes are shown in Figure 7. RT-qPCR was performed to assess the expression level of target genes expression level in mutant plants and WT. The rice *actin* gene was used as internal control. The RT-qPCR results exhibited that the expression of *GS3* was significantly suppressed in mutant plants compared to WT (Figure 7A). To validate the proteomic data, we selected the five genes associated with DEPs. In total, two key genes encoding down-regulated proteins including *Os01g0270100* and *GLUP6*, and three genes encoding up-regulated proteins including *Os01g0978100*, *Os06g0698859*, and *Os06g0698859* were chosen. The protein expression pattern obtained by iTRAQ differential labeling was consistent with the trend of mRNA expression pattern in RT-qPCR (Figure 7B), indicating that the result of identifying and quantifying the proteome by iTRAQ differential labeling combined with high-performance liquid chromatography is reliable. The primers used for RT-qPCR are enlisted in Table S5.

## 3. Discussion

Gene-editing technology has emerged in recent years, which uses sequence-specific nucleases (SSNs) to introduce or delete bases at specific sites in DNA to generate high-frequency-induced mutations in target genes. It has attracted the attention of most researchers because of its broad application prospects in the fields of medicine, agriculture, and industry. Compared with conventional breeding, CRISPR/Cas9 technology is widely used in rice germplasm improvement due to its simple operation, high editing efficiency, and low cost [11,12,28,31], which greatly shortens the breeding cycle. CRISPR/Cas9 has been widely used for crop genetic improvement, and various vectors have been developed which are used to edit the genome of monocots and dicots [24]. The mining and utilization of key functional genes is an important way to improve crop yield and quality, but obtaining mutants with loss of function is a prerequisite for the identification of genetic functions of genes. Traditional genetic research strategies, such as EMS mutagenesis, T-DNA insertion mutation, transposon insertion mutation, and RNA interference greatly limit the functional identification of target genes due to the random nature of mutation sites. The CRISPR/Cas gene-editing technology has the advantages of high efficiency, and it can knock out almost any functional gene. Therefore, it has shown great application prospects in the field of research on the utilization of key functional genes for crop improvement. Currently, rice is one of the most successful crops for CRISPR/Cas9 applications. Studies have shown that in CRISPR/Cas9-mediated T_0_ mutants, the probability of obtaining homozygous or biallelic mutations is highest [10,11,12]. 

The editing results of the *GS3* gene showed that the *Cas9* system with a U6 promoter had high efficiency. The editing efficiency of target one was 73%, and the editing efficiency of the second target was 80%. Currently, CRISPR/Cas9 is widely used in rice, and an editing efficiency of more than 80% has been achieved [30]. Higher editing efficiency makes it easy to get a variety of different types of mutations. In this study, the deletions were occurred frequently, whereas the rate of base insertion was relatively low. The GL and GWT were increased by 31.39% and 27.15%, respectively. The present results show that the use of CRISPR/Cas9 technology can breed long-grain and high-yielding varieties and accelerate the creation of long-grain germplasm resources.

To identify the proteome-wide changes between mutants and WT, a comparative iTRAQ-proteomics analysis was performed, and DEPs were screened successfully. We found that proteins related to cysteine synthase, cysteine proteinase inhibitor, ubiquitin, vacuolar protein sorting-associated, and DNA ligase were differentially regulated in mutant plants. The GO analysis also showed that DEPs were enriched in cellular process, metabolic process, binding, transmembrane, structural and catalytic activities. The KEGG analysis found that the DEPs were only enriched in lipid metabolism and oxylipin biosynthesis. In the PPI network, the proteins related to DNA damage-binding, JUN-activation-domain-binding, vacuolar protein sorting-associated, ubiquitin-40S ribosomal, and cysteine proteinase inhibitor showed the highest interaction.

The plant ubiquitin/proteasome system is the main way of protein degradation in cells and plays an important role in the process of plant growth and development, morphogenesis, and disease resistance [32]. Recent studies have shown that some pathogenic bacteria can simulate the host plant ubiquitin/protease system components [33]. The ubiquitin/proteasome pathway is mainly composed of ubiquitin-activating enzyme E1, ubiquitin-conjugating enzyme E2, ubiquitin-protein ligase E3, proteasome, and deubiquitinating enzymes (DUBs). Studies have shown that ubiquitination plays an important role in plant growth and development and the response of plants to biotic and abiotic stress [33,34,35]. Previous studies revealed that ubiquitin ligase is a functional partner of the Ribosome Quality Control Complex (RQC), which forms a stable complex with 60S ribosomal subunits containing stalled polypeptides and triggers their degradation. It also causes dissociation of the ribosome into the 40S and 60S subunits and translation-stress signaling pathway from the ribosome dependent on the RQC members [36]. Ubiquitin has been characterized as a mark for the degradation of proteins and regulates endogenous proteins lacking any folding defect [37,38,39]. Ubiquitin can control various cellular processes by altering protein localization, inducing structural changes, and regulating protein interactions [40,41,42,43]. These different functions have been observed in all signaling pathways, such as DNA repair, endocytosis, kinase regulation, transcriptional and translational control [43,44,45,46]. The Arabidopsis E3 ubiquitin ligase positively regulates the cytosol’s protein levels, probably before importing pre-proteins into chloroplast during the formation of functional chloroplasts [47]. Misfolded proteins exported to the cytosol are subsequently ubiquitinated by E1 ubiquitin-activating enzymes, E2 ubiquitin-conjugating enzymes, and E3 ubiquitin ligases for their eventual degradation through the 26S proteasome [48,49]. The cytosolic protein recognizes specific sequence motifs and promotes pre-protein degradation by the 26S proteasome through interaction with the C-terminus of the E3 ubiquitin ligase Hsc70-interacting protein in Arabidopsis [50].

The results indicate that *GS3* mutations may function in the ubiquitination pathway. Interestingly, among the four grain-weight related genes identified previously through map-based cloning of QTLs, three of them encoding proteins that are possibly associated with the ubiquitination types of machinery. *GW2* has been shown to encode a cytosolic RING-type protein with E3 ubiquitin ligase activity [14], and *GW5/qSW5* encodes a nuclear protein that physically interacts with a polyubiquitin [51,52], whereas the protein encoded by *GS3* contains a putative cysteine-rich domain of the tumor necrosis factor receptor (TNFR), which likely colocalizes with ubiquitin in human cells [53]. These data together raise the possibility that *GS3*, *GW2*, and *GW5*/*qSW5* act through the same ubiquitination pathways to determine grain size and grain weight in rice.

Cysteine is a superfamily of secreted proteins that are widely found in plants and animals. Its primary function is to interact with proteases and control various physiological traits of organisms. These proteins are involved in preventing the functional protein from being degraded by exogenous proteases. The cysteine protease inhibitor superfamily can be divided into three families (I, II, and III) according to the amino acid sequence [54,55,56]. Cysteine proteins are recognized as the major enzymes for the catabolism of the majority of reserve proteins in seeds. Studies have shown that plant cysteine proteinase inhibitor proteins play an important role in terms of insect feeding inhibitors, resulting in abnormal development or death of insects [57,58,59,60,61]. In addition, cysteine protease inhibitors are also involved in plant growth and development, such as seed germination and maturation, seedling growth, fruit ripening, and programmed cell death [62,63,64,65] and improving plant abiotic stress tolerance [66,67,68]. At present, cysteine protease inhibitors have been cloned from a variety of plants, such as *Arabidopsis thaliana* [57], rice [69], cotton [70], and tobacco [71]. Vacuolar protein sorting-associated (VPS) proteins are the main component of endosomal sorting and transport complex II and play an important role in the ubiquitin-mediated degradation of membrane proteins in the multivesicular pathway. In rice, *OsVPS22* is very important for seedling viability and grain filling [72]. Lipid metabolism plays a vital role in plant reproductive development and is also involved in biosynthesis and transport [73,74]. Lipid metabolism is also crucial for callus formation, female gametophyte development, spikelet development, and flowering [75,76,77,78,79]. The defects in lipid metabolism-related genes are lethal to embryo or seed development in various plants [80,81,82]. Lipid-derived jasmonic acids (JA) genes have been demonstrated to play critical roles in anther dehiscence and pollen maturation in Arabidopsis and rice. Loss functions of genes, such as *dad1*, *fad3*/*fad7*/*fad8*, *opr3*, and *aos* in Arabidopsis, and *osjar1* in rice, generally lead to defects in anther dehiscence, filament elongation, and pollen maturation [83,84,85,86,87]. Several JA biosynthetic genes, *Defective in Anther Dehiscence1*/*Extra Glume 1 (OsDAD1/EG1)*, *Allene Oxide Cyclase gene (OsAOC)*, *Jasmonic Acid Carboxyl Methyltransferase gene (OsJMT)*, and *Open Glume 1 (OsOG1)*/*OsOPR7* in rice, are required for rice spikelet development and flowering [77,88,89,90]. Therefore, it might increase grain yield per plant by proper manipulation of these genes in rice. Notably, the mechanism of lipid metabolism underlying GS3 mutations and reproductive development processes needs to be further elucidated.

We can attribute that functional crosstalk maybe exists between the differentially regulated proteins. Thus, different functional changes generated by the mutations analyzed here could directly and/or indirectly contribute to the mutant’s phenotype. Alternatively, the relatively increased level of expression of these proteins may be advantageous in grain development. The differential expression of these DEPs may be triggered by *GS3* mutations. It is speculated that *GS3* mutations may regulate the expression of vacuolar protein sorting-associated, putative ubiquitin, and cysteine proteinase inhibitor proteins, and then play a positive regulatory role in the process of grain development. Therefore, the molecular mechanism of the differential response of these proteins in *GS3* mutants deserves further attention. The author believes that using different bioinformatic approaches and gene functional studies, it is possible to further understand the molecular regulation network mechanism of rice grain development to select parents with improved yield.

## 4. Materials and Methods

### 4.1. Test Materials

**Figure 8:**
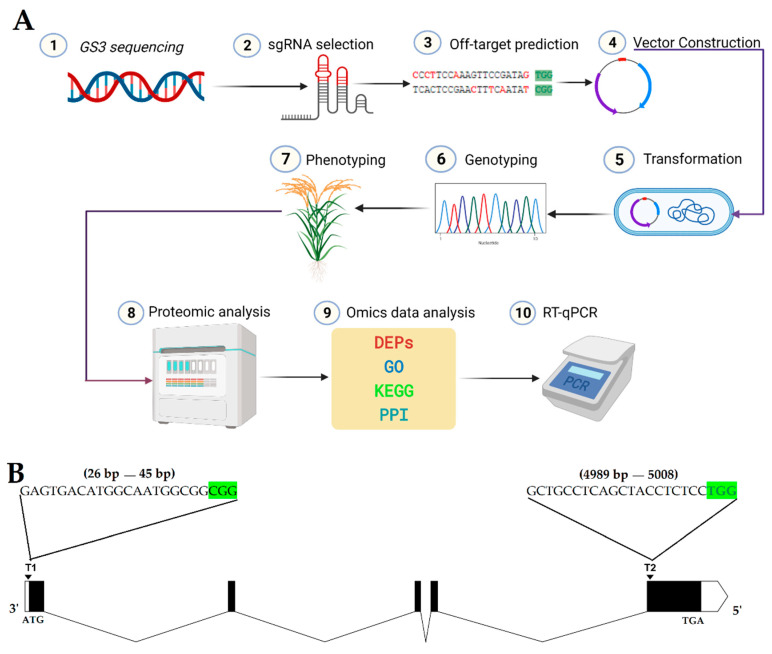
(**A**) Schematic diagram of the procedure for CRISPR/Cas9-based generation of mutant plants and analysis of mutations. Two sgRNAs were selected using the CRISPR-GE online web-based tool, and vector was constructed. Agrobacterium-mediated transformation was performed, and T_0_ plants were regenerated. Later generations were produced by self-pollination, and genotyping was performed using target-specific primers. The phenotypic data of mutant and wild-type (WT) plants were recorded and further analyzed. The proteomic analysis was also performed, and RT-qPCR was performed to assess the *GS3* expression level and validate the proteomic data. (**B**) Diagram of *GS3* gene and positions of both target sites. ATG is the start codon; TGA is the stop codon; green highlighted CGG and TGG are the PAM sequences; the white boxes at extreme left and right represent the untranslated (UTR) regions, the black boxes represents the exons; black lines in between the exon regions represent the intron regions, T1 and T2 represent target 1 and target 2, respectively.

This study used the japonica rice variety TP309 as the test material. WT and mutant plants were grown in the experimental area of Guangxi University (45° N latitude) and Hainan (19° N latitude) and planted with a row spacing of 25 cm × 25 cm under natural conditions. The promoters (U6a and U6b) and pYLCRISPR/Cas9Pubi-H binary vector used in the experiment were kindly provided by Liu Yaoguang, South China Agricultural University. In this study, the CRISPR/Cas9 construct was carefully designed with higher specificity and low off-target score, and the *GS3* gene with the expectation to produce a null mutation was edited. Moreover, the iTRAQ-based proteomic analysis was also performed to assess the effect of mutations on the whole proteome. The schematic representation of the entire workflow of generation and analysis of targeted mutated plants is described in Figure 8A.

### 4.2. gRNA Design, Vector Construction, and Transformation

The gene sequence of *GS3* (*Os03g0407400*) was retrieved through NCBI (https://www.ncbi.nlm.nih.gov/) [91] and using the CRISPR-GE online website (http://skl.scau.edu.cn/) [92], a pair of targets and their linker primers were designed in the first and last exon (Figure 8B; Table S6). The structure of both sgRNAs was developed using CRISPR-P ver 2.0 (http://crispr.hzau.edu.cn/CRISPR2/) [93] (Figure S2). Referring to Ma et al. (2015) [94], the vector was contrasted. The connected vector was transferred into DH5α by the heat shock method. The positive colonies were selected by colony PCR using specific primers (SPL1/SP-R), and the product was directly sequenced. The plasmid that was detected correctly was transferred into EHA105 Agrobacterium competent cells by heat shock. EHA105 by electroporation and rice transformation was performed according to Hiei et al. (1994) [95].

### 4.3. Genotyping, Phenotyping, and Screening of T-DNA-free Plants

At the seedling stage of the T_0_ plants, the CTAB method was used to extract the genomic DNA from the leaves, and the target-specific primers (GS3T1F/R and GS3T2F/R) were used for genotyping. The sequencing results were detected using the degenerate sequence decoding (DSDecode) method [96] to analyze the mutation types and frequency. The leaf DNA of the transgenic T_0_ generation plants was extracted by the CTAB method and stored in a refrigerator at −20 °C. The designed hygromycin primer HPTF/R and carrier primer Cas9F/R were used to screen T-DNA-free plants. The PCR system was as follows: 2× *Taq* Master Mix 7.5 μL, forward and reverse primers (10 μ mol/L) 0.5μL each, 1 μL DNA, 5.5 μL ddH_2_O. The PCR program was as follows: 1 min and 30 s at 94 °C; 30 s at 94 °C; 30 s at 57 °C; 30 s at 72 °C; 5 min at 72 °C; and 5 min at 10 °C. The PCR products were run on gel electrophoresis using 2% agarose gel and stained with ethidium bromide solution. The plants not showing bands were considered as T-DNA-free plants. WT, T_0_, T_1_, and T_2_ generation plants were planted in field conditions, and the agronomic data were recorded. The PH, FLL, and GLW were measured at the maturity stage. The seeds were dried, and data for GL, GWD, and GWT were measured from randomly selected grains.

### 4.4. Protein Extraction and Quality Inspection

The three g of rice leaf samples from WT and mutant plant of GXU27-4 were taken and ground into a powder with liquid nitrogen and transferred to a 15 mL centrifuge tube. Protein extraction buffer (8 mol/L urea, 0.1% SDS, 1 mmol/L PMSF, 1 mmol/L DTT) was added to the sample and shaken for 3 h at room temperature on an automatic vortex mixer (Thermo Fisher Scientific, Shanghai, China). After vortexing, the sample was centrifuged for 15 min at 4 °C, 14,000 r/min, and the supernatant was transferred to a new 15 mL centrifuge tube. Pre-cooled acetone was added to the 6 times volume of a sample and precipitated overnight at −20 °C. The precipitated overnight sample was centrifuged at 4 °C and 12,000× *g* for 15 min, and the supernatant was removed. The precipitate was vacuum dried at 4 °C to obtain the total protein dry powder, which was stored at −80 °C for later use.

A total of 50 mg of dry protein powder was taken and lysis solution (containing 8 mol/L urea, 2 mol/L thiourea, mass fraction 4% CHAPS, pH 3–10 volume fraction 0.5% ampholyte, 50 mmol/L dithiothreitol (DTT), and 1.0 mmol/L after phenylmethanesulfonyl fluoride (PMSF)) was added, dissolved, and centrifuged at 4 °C, 12,000× *g* for 15 min. The supernatant (total protein solution) was transferred to a 1.5 mL centrifuge tube and SDS-PAGE electrophoresis, and Coomassie brilliant blue staining were used to detect the integrity of the total protein. The concentration of protein samples was determined by the Bradford protein assay.

### 4.5. Protein Digestion and iTRAQ Labeling

The protein sample was first reduced and alkylated and then digested with trypsin enzyme. One hundred μg of protein was taken from each sample, and after incubating with 10 mmol/L dithiothreitol (DTT) at 37 °C for 1 h, and 55 mmol/L iodoacetamide at room temperature for 1 h, 3.3 μg of pancreatic protein enzyme digestion was carried out at 37 °C for 12 h. At the end of digestion, 100 μL of formic acid with a volume fraction of 1% was added to terminate the enzymatic hydrolysis reaction and then vacuum-dried to obtain a dry powder of protein peptides. The dry powder of protein peptides was dissolved in 8 mol/L urea (containing 0.1% SDS) and 500 mmol/L triethylammonium bicarbonate in water and the 8-plex iTRAQ kit of AB Sciex was used for labeling. The 6 tubes of labeling reagents (110, 111, 112, 113, 114, and 115) in the kit were diluted with 50 μL of isopropanol and mixed with the corresponding protein-peptide dry powder reconstitution samples and placed at room temperature. The three WT and GXU27-4 samples were labeled with iTRAQ reagent.

### 4.6. Strong Cation Exchange Column Classification and Mass Spectrometry Detection of iTRAQ Labeled Samples

Six iTRAQ-labeled samples were mixed in equal amounts and then graded using a C18 strong cation column (SEC). The peptide mixture was loaded onto SEC in equilibration buffer A (containing pH 2.55, 5 mmol/L KH_2_PO_4_, 20% acetonitrile, and H_3_PO_4_ by volume) for 25 min. Then, 300 μL of the labeled sample mixture was taken and used SEC equilibration buffer B (containing pH 2.75, 5 mmol/L KH _2_PO_4_, 20% acetonitrile by volume, 600 mmol/L KCl, H _3_PO_4_) to dilute 7 times, and orthophosphoric acid was added to adjust the pH value to 2.5. The sample was centrifuged to take the supernatant for gradient elution with the eluent flow rate of 0.2 mL/min. In the full scan, the top 20 precursor ions of the ion intensity were selected after fragmentation in the higher-energy collisional dissociation (HCD) mode with a standard collision energy of 30 eV, and then, the secondary mass spectrometry sequence was determined to report the ion. The rest of the procedure was followed according to Wang et al. (2014) [97].

### 4.7. Proteomic Data Analysis and Functional Annotation

The mass spectrometry results were imported into the Proteome Discoverer software to search the Uniprot database (http://www.uniprot.org/) for the functional annotation of the protein. According to the protein expression counted by the software Proteome Discoverer, the DEPs analysis was performed. The DEPs were screened between the WT and mutant lines with a fold change (FC) of 1.2 and *p* < 0.05. The online GO database (http://geneontology.org/) and the KEGG database (http://www.genome.jp/kegg/pathway) were searched using the KOBAS software according to the protein-coding gene ID. STRING database (version 10.0) (https://string-db.org/) was searched for PPI network for all up and down-regulated proteins. Then, the network was visualized by Cytoscape version 3.8.0.

### 4.8. Verification of mRNA Expression of GS3 and Protein-Coding Genes

The expression pattern of *GS3* was analyzed in WT and mutants’ plants. To verify the reliability of the iTRAQ data, 10 representative DEPs were selected from different functional classifications. According to their coding genes, the mRNA sequences were retrieved from China Rice Data Center (http://www.ricedata.cn/), and RTqPCR primers were designed using qPrimerDB (https://biodb.swu.edu.cn/qprimerdb/). The RT-qPCR analyses were conducted, and the gene expression was calculated by using the 2^−ΔΔ*C*t^ (cycle threshold) process as described previously [98].

### 4.9. Statistical Analyses

Statistical analysis (*p* < 0.05) was completed using SPSS 16.0 Statistical Software Program and GraphPad Prism (version 7.0, GraphPad Software Inc. San Diego, CA, USA) was used to display the graphs.

## 5. Conclusions

In this study, *GS3* mutants were generated using the CRISPR/Cas9 system and iTRAQ-based quantitative proteomic analysis was performed. The proteome-wide characterization of mutants provides new understandings, and the targeted genome editing also facilitated the identification DEPs and pathways that may be involved in rice grain development. The differential response of proteins related to cysteine synthase, cysteine proteinase inhibitor, ubiquitin, and DNA ligase in *GS3* mutants will provide a reference and sharp focus in rice molecular breeding. Therefore, the molecular mechanism of the up-regulation of these proteins’ expression in *GS3* mutants deserves further attention. Genome editing facilitated pathway-level study, and DEPs found in this study might be directly or indirectly involved in rice grain development and can be analyzed further to reveal their functional role. The mutants with different mutations represent population diversity, which is the main driving force of breeding work. This study provides a strategy for the rapid introduction of genetic diversity in the process of crop breeding and practical significance.

## Figures and Tables

**Table 1:** Increased yield of mutant lines in T_0_, T_1_, and T_2_ generations.

Generation	Genotypes	PH	PN	FLL	FLW	GNPP	GL	GWD	GWT
**T_0_**	WT	145.3 ± 3.6	9.5 ± 1.6	60.2 ± 2.3	2.6 ± 0.3	148.6 ± 0.9	8.7 ± 0.2	2.9 ± 0.1	31.3 ± 1.3
	GXU27-3	144.6 ± 4.2 ^ns^	9.7 ± 2.2 ^ns^	59.9 ± 3.1 ^ns^	2.5 ± 0.5 ^ns^	147.4 ± 0.8 ^ns^	11.1 ± 0.3 *	3.0 ± 0.3 ^ns^	39.8 ± 1.2 *
	GXU27-4	146.4 ± 5.6 ^ns^	9.6 ± 1.4 ^ns^	60.3 ± 2.2 ^ns^	2.6 ± 0.4 ^ns^	146.5 ± 0.7 ^ns^	10.8 ± 0.5 *	2.9 ± 0.2 ^ns^	39.1 ± 1.6 *
	GXU27-9	145.9 ± 4.8 ^ns^	9.7 ± 1.8 ^ns^	59.8 ± 2.5 ^ns^	2.7 ± 0.3 ^ns^	149.2 ± 0.8 ^ns^	10.9 ± 0.2 *	2.9 ± 0.1 ^ns^	39.0 ± 1.4 *
**T_1_**	WT	146.5 ± 2.8	9.6 ± 1.5	61.3 ± 2.6	2.5 ± 0.2	149.4 ± 0.6	8.8 ± 0.3	3.0 ± 0.1	32.1 ± 1.1
	GXU27-3	145.4 ± 3.4 ^ns^	9.8 ± 1.9 ^ns^	60.2 ± 2.4 ^ns^	2.7 ± 0.2 ^ns^	148.5 ± 0.9 ^ns^	11.3 ± 0.2 *	3.1 ± 0.1 ^ns^	39.7 ± 1.4 *
	GXU27-4	147.3 ± 4.2 ^ns^	9.8 ± 1.2 ^ns^	59.9 ± 2.5 ^ns^	2.4 ± 0.5 ^ns^	148.2 ± 0.5 ^ns^	10.9 ± 0.3 *	3.0 ± 0.2 ^ns^	39.3 ± 1.0 *
	GXU27-9	145.8 ± 3.5 ^ns^	9.6 ± 1.4 ^ns^	60.1 ± 2.2 ^ns^	2.6 ± 0.4 ^ns^	149.6 ± 0.4 ^ns^	10.6 ± 0.4 *	2.9 ± 0.2 ^ns^	39.2 ± 1.2 *
**T_2_**	WT	144.6 ± 2.8	9.8 ± 1.3	59.9 ± 2.4	2.7 ± 0.2	148.2 ± 0.6	8.6 ± 0.3	2.9 ± 0.2	31.6 ± 1.2
	GXU27-3	144.8 ± 3.7 ^ns^	9.6 ± 2.0 ^ns^	60.1 ± 3.2 ^ns^	2.6 ± 0.3 ^ns^	145.4 ± 0.6 ^ns^	11.2 ± 0.4 *	3.0 ± 0.1 ^ns^	39.6 ± 1.5 *
	GXU27-4	145.9 ± 3.5 ^ns^	9.8 ± 1.6 ^ns^	60.0 ± 2.3 ^ns^	2.6 ± 0.4 ^ns^	147.5 ± 0.5 ^ns^	10.9 ± 0.3 *	3.0 ± 0.1 ^ns^	39.3 ± 1.2 *
	GXU27-9	145.3 ± 4.2 ^ns^	9.9 ± 1.7 ^ns^	59.9 ± 2.4 ^ns^	2.5 ± 0.2 ^ns^	149.3 ± 0.7 ^ns^	10.7 ± 0.2 *	2.9 ± 0.3 ^ns^	39.1 ± 1.3 *

WT (wild type); PH (plant height) cm; PN (panicle numbers); FLL (flag leaf length) cm; FLW (flag leaf width) cm; GNPP (grain number per panicle); GL (grain length) mm; GWD (grain width) mm; GWT (1000-grain weight) g. Five independent plants were used to collect data from three replicates (n = 5). For grain phenotyping, five grains from each plant were selected randomly. * and ^ns^ denote the significant and non-significant differences (Student’s *t*-test, *p* < 0.01), respectively.

**Table 2:** List of some important differentially expressed proteins (DEPs).

Protein ID	Protein Names	log2 FC	Regulation
A2ZMY2	Cysteine synthase	1.66	Up
Q5JNB0	Cysteine synthase	1.55	Up
B8AJV7	Cysteine synthase	1.46	Up
Q0JNR2	Cysteine proteinase inhibitor 12	−2.38	Down
A0A0A7EQF3	Cysteine proteinase inhibitor	−2.66	Down
P20907	Cysteine proteinase inhibitor 2	−2.30	Down
A2XEA1	Ubiquitin	−1.50	Down
Q10NQ3	Vacuolar protein sorting-associated protein 9A	2.87	Up
A2X377	Vacuolar protein sorting-associated protein 29	3.12	Up
A0A0E0GXY5	Vacuolar protein sorting-associated protein 41 homolog	3.09	Up
Q8H8K1	Putative vacuolar sorting receptor protein	2.13	Up
Q6L4S0	DNA damage-binding protein 1	1.86	Up
Q7XD67	DNA ligase	3.83	Up

## Data Availability

Raw data can be provided to researchers interested on request to the corresponding author.

## Supplementary Materials

The following are available online at https://www.mdpi.com/1422-0067/22/6/3225/s1.

## Abbreviations

CRISPR	Clustered regularly interspaced short palindromic repeats
Cas9	CRISPR-associated protein 9
GWT	1000-grain weight
GL	Grain length
iTRAQ	Isobaric tags for relative and absolute quantitation
DEPs	Differentially expressed proteins
KEGG	Kyoto Encyclopedia of Genes and Genomes
GO	Gene Ontology
