# Supplementary material for: CRISPR/Cas9 Guided Mutagenesis of Grain Size 3 Confers Increased Rice (Oryza sativa L.) Grain Length by Regulating Cysteine Proteinase Inhibitor and Ubiquitin-Related Proteins
Source: Int J Mol Sci. 2021 Mar 22;22(6):3225. doi: 10.3390/ijms22063225 (PMC8004693; doi:10.3390/ijms22063225)
Supplement: Supplementary file 1 [file ijms-22-03225-s001.zip › ijms-1148941-suppl/Figures and Tables.docx]

**Table S1.** List of primers used for the construction of vector and genotyping of mutant plants.

| **Primer name** | **Primer Sequence (5'-3')** |
| --- | --- |
| GS3T1F/R | F:TCAGTTCCCCAAAAACTGCT |
|  | R:TGAACAGTCAACAGGGATCG |
| GS3T2F/R | F:GTGCATGATGCTTTCACCAC |
|  | R:TTGAGGTTGAAGGAGGAGGA |
| gRT1: | AGTGACATGGCAATGGCGGgttttagagctagaaat |
| OsU6aT1: | CCGCCATTGCCATGTCACTCggcagccaagccagca |
| gRT2: | CTGCCTCAGCTACCTCTCCgttttagagctagaaat |
| OsU6aT2: | GGAGAGGTAGCTGAGGCAGCaacacaagcggcagc |
| U-F | CTCCGTTTTACCTGTGGAATCG |
| gR-R | CGGAGGAAAATTCCATCCAC |
| Pps-R | TTCAGA**ggtctc**T**ACCG**ACTAGTATGGAATCGGCAGCAAAGG |
| Pgs-2 | AGCGTG**ggtctc**G**tcag**ggTCCATCCACTCCAAGCTC |
| Pps-2 | TTCAGA**ggtctc**T**ctga**cacTGGAATCGGCAGCAAAGG |
| Pgs-L | AGCGTGGGTCTCGTCTTCACTCCATCCACTCCAAGCTC |
| PB-R | GCGCGCGGTCTCTACCGACGCGTATCC |
| PB-L | GCGCGCgGTCTCGCTCGACTAGTATGG |
| HPT | F: GTGCTTGACATTGGGGAGTT  R: ATTTGTGTACGCCCGACAGT |
| Cas9-F | CTGACGCTAACCTCGACAAG |
| Cas9-R | CCGATCTAGTAACATAGATGACACC |
| SP-L1 | GCGGTGTCATCTATGTTACTAG |
| SP-R | GCCTATACCAAGTTATTGCA |

**Table S2.** Primers designed for off-target sites evaluation.

| **Primer name** | **Primer Sequence (5'-3')** |
| --- | --- |
| POT1 | F: ATAAACCGGGGTGAACTTCC  R: GTTGCTCGTGAGAGGGAGAG |
| POT2 | F: CAGGCACTCATCGAGCTACA  R: AGATCATGCCGTCGTTCTG |
| POT3 | F: GCCTTTGGCATTAGATTGGA  R: GCAGTCCACCACTCTTCCTC |
| POT4 | F: AAATGAATTCGGCTTGATCG  R: GCAGGTACACCGCCTCTATC |
| POT5 | F: TCTTTGTGCTCACCGAACTG  R: TGGAAAAGATGCAGCAGATG |
| POT6 | F: CTCGGGTACTTCCTCAGCAC  R: TGGCCCAGAAGAGGTAGTTG |
| POT7 | F: ACGTAGGCCACAAGGATGAC  R: AGAGGAGGGGGATGTACCTG |
| POT8 | F: CGTGGCGCTACTTCTCCTT  R: GCCAGAGGTAGACGAGATGC |
| POT9 | F: GTGCTGTCGCTCTCGTCTC  R: GTCCTGGTGGTGGTGTTCAT |
| POT10 | F: GTGCTGTCGCTCTCGTCTC  R: GTCCTGGTGGTGGTGTTCAT |

**Table S3.** Mutations detection on five most likely putative off-target sites.

| **Target** | **NOPO** | **GL** | **Locus** | **Sequence** | **MMB** | **NPS** | **NPOM** | **OTS** | **Region** |
| --- | --- | --- | --- | --- | --- | --- | --- | --- | --- |
| T1 | OT1 | Chr6: 7987883-7987905 | LOC_Os06g14324 | GAGCAACAGGGCAATGGCGG CGG | 3 | 20 | 0 | 0.347 | 5’UTR |
|  | OT2 | Chr4: 5006091-5006113 | LOC_Os04g09390 | GAAGGCCATGGCGATGGCGG CGG | 4 | 20 | 0 | 0.139 | CDS |
|  | OT3 | Chr10: 818889-818911 | LOC_Os10g02284 | GAGTGACATGGCAATGAGGT CGG | 3 | 20 | 0 | 0.087 | CDS |
|  | OT4 | Chr7: 23852331-23852353 | LOC_Os07g39810 | GCTTGCGATGGCAATGGCGG CGG | 4 | 20 | 0 | 0.084 | CDS |
|  | OT5 | Chr9: 5157069-5157091 | LOC_Os09g09550 | GACGGAGGTGGCAATGGCGG CGG | 4 | 20 | 0 | 0.083 | CDS |
| T2 | OT6 | Chr1: 31358649-31358671 | LOC_Os01g54515 | ACTTCCTCAACTACCTCTTC TGG | 4 | 20 | 0 | 0.141 | CDS |
|  | OT7 | Chr11: 28648206-28648228 | LOC_Os11g47446 | GCTGCCTACGCTACCTCGCC GGG | 3 | 20 | 0 | 0.124 | CDS |
|  | OT8 | Chr9: 21313672-21313694 | LOC_Os09g36940 | TCGGCCCCAGCTACCTCGCC TGG | 4 | 20 | 0 | 0.115 | CDS |
|  | OT9 | Chr9: 908085-908107 | LOC_Os09g02250 | GCTGCCTCTTCTTCCTCACC TGG | 4 | 20 | 0 | 0.098 | CDS |
|  | OT10 | Chr4: 24546876-24546898 | LOC_Os04g41400 | GCTTCGTCAACTACCTCTAC TGG | 4 | 20 | 0 | 0.073 | CDS |

Note: The protospacer adjacent motif (PAM) (NGG) is shown in green background. NOPO; name of putative off-target, GL; genomic location, MMB; mis-matching bases, NPS; number of plants screened, NPOM; number of plants with off-target mutations, OTS; off-target score. T1 and T2 represents target1 and target2.

**Table S4.** Segregation pattern of homozygous, mono-allelic heterozygous, and bi-allelic heterozygous mutations in T_1_ generation.

| **Targets** | **T_0_** | | | **T_1_** | | | | |
| --- | --- | --- | --- | --- | --- | --- | --- | --- |
|  | **Mutants** | **Zygosity** | **InDels** | **PT** | **WT** | **Bi** | **Homo** | **Hetero** |
| T1 | GXU27-4 | Hom | 25d/25d | 25 | 0 | 0 | 25(25d/25d) | 0 |
| T2 | GXU27-4 | Hom | 6d/6d | 25 | 0 | 0 | 25(6d/6d) | 0 |
| T1 | GXU27-3 | Mono Het | 3d/- | 43 | 11 | 0 | 22(3d/3d) | 10(3d/-) |
| T2 | GXU27-8 | Mono Het | 4d/- | 43 | 13 | 0 | 21(4d/4d) | 9(4d/-) |
| T1 | GXU27-1 | Bi Het | 1d/1i | 43 | 0 | 21(1d/1i) | 12(1d), 10(1i) | 0 |
| T2 | GXU27-2 | Bi Het | 2d/1i | 43 | 0 | 22(2d/1i) | 10(2d), 11(1i) | 0 |

PT; number of plants tested, WT; wild type, Bi Het; bi-allelic heterozygous, Homo; homozygous, Mono Het; mono-allelic heterozygous, Chi; chimeric. d: deletion, i: insertion and WT: wild type. The numbers in front of the letters indicate the number of nucleotides affected. Corresponding mutations in two alleles are distinguished by ‘/’.

**Table S5.** Primers designed for RT-qPCR analysis.

| **Gene ID** | **Forward Primer (3’-5’)** | **Reverse Primer (5’-3’)** |
| --- | --- | --- |
| *GS3* | AAGTCACGTGTGGAATCCCT | GAGTTGTTCGTTGCTGGTGT |
| *Os01g0978100* | TGCGCGTTACCTACCTACTT | GGGCGTATCTACTCAGCCTT |
| *Os01g0270100* | AAGAAGGTGTACGAGGCCAA | GTTGGTGAAGGTGGTTGCAT |
| *Os06g0698859* | AGACCCTCTTTCTGGTGCTC | GTCAACTTGAACCCGTCGAG |
| GLUP6 | TTACGTTCCCGTGTCCTTCA | CCTCGGAACTACACAGACGA |
| *Os05g0494200* | ATCATTGGCAGGAGGCTGAT | ATCACTGGATGGCTCTGCTT |

**Table S6.** Selected target positions with their GC content and potential off-target score.

| **Target** | **Target Sequence (5’-3’)** | **Position** | **Strand** | **GC %** | **Region** | **Off-target Score** | **Pairing with sgRNA (>=8 nt)** |
| --- | --- | --- | --- | --- | --- | --- | --- |
| T1 | GAGTGACATGGCAATGGCGGCGG | 26-45 | + | 60.0 | CDS | 0.347 | None |
| T2 | GCTGCCTCAGCTACCTCTCC**TGG** | 4989-5008 | + | 65.0 | CDS | 0.18 | None |

T1 and T2; represent the Target1 and Target2. Green highlighted are PAM regions.


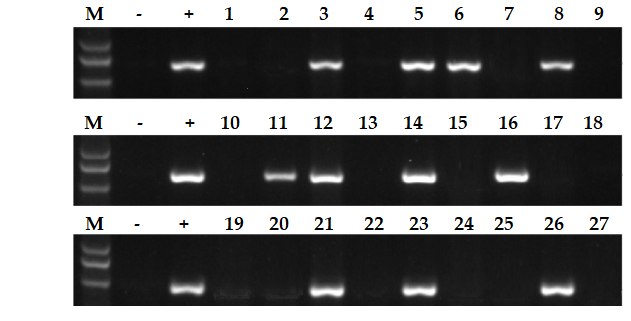


**Figure S1.** Screening of Transgene (T-DNA) free plants using Cas9 specific primers. Two randomly selected plants from each mutant line were evaluated. The mutant lines that failed to amplify to the target sequence were termed as T-DNA-free. M, Marker D5000; +, Positive control; -, Negative control; 1 to 27 numbers represents mutant plants.

**
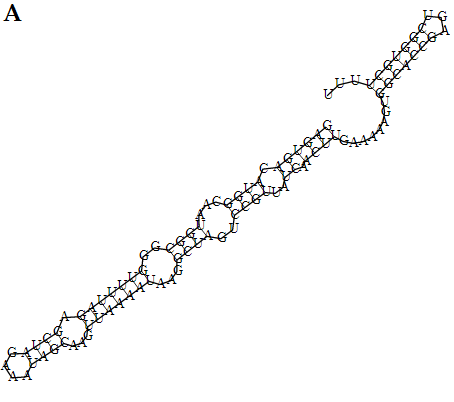

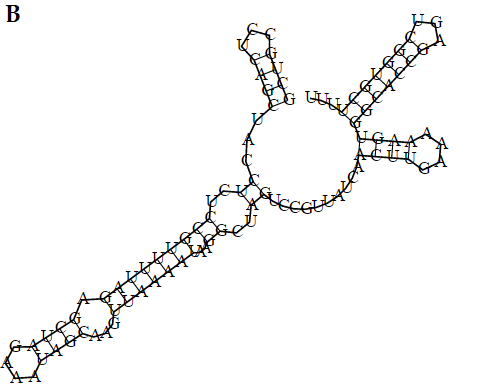
**

**Figure S2.** Schematic representation of secondary structures of **(A)** sgRNA1; and **(B)** sgRNA2; used in the experiment.
